# Ketogenic diet does not affect strength performance in elite artistic gymnasts

**DOI:** 10.1186/1550-2783-9-34

**Published:** 2012-07-26

**Authors:** Antonio Paoli, Keith Grimaldi, Dominic D’Agostino, Lorenzo Cenci, Tatiana Moro, Antonino Bianco, Antonio Palma

**Affiliations:** 1 Physiological Laboratory – Department of Biomedical Sciences, University of Padova, Padova, Italy; 2 Human Movement Sciences School, University of Padova, Padova, Italy; 3 Biomedical Engineering Laboratory, Institute of Communication and Computer Systems, National Technical University of Athens, Athens, Greece; 4 Department of Molecular Pharmacology & Physiology College of Medicine, University of South Florida, Tampa, FL, USA; 5 Tisanoreica Study Center, Lonigo, Vicenza, Italy; 6 Department of Sports and Exercise Science (DISMOT), University of Palermo, Palermo, Italy

**Keywords:** Very low carbohydrate Ketogenic diet, Body composition, Weight loss, Strength, Gymnastic

## Abstract

**Background:**

Despite the increasing use of very low carbohydrate ketogenic diets (VLCKD) in weight control and management of the metabolic syndrome there is a paucity of research about effects of VLCKD on sport performance. Ketogenic diets may be useful in sports that include weight class divisions and the aim of our study was to investigate the influence of VLCKD on explosive strength performance.

**Methods:**

8 athletes, elite artistic gymnasts (age 20.9 ± 5.5 yrs) were recruited. We analyzed body composition and various performance aspects (hanging straight leg raise, ground push up, parallel bar dips, pull up, squat jump, countermovement jump, 30 sec continuous jumps) before and after 30 days of a modified ketogenic diet. The diet was based on green vegetables, olive oil, fish and meat plus dishes composed of high quality protein and virtually zero carbohydrates, but which mimicked their taste, with the addition of some herbal extracts. During the VLCKD the athletes performed the normal training program. After three months the same protocol, tests were performed before and after 30 days of the athletes’ usual diet (a typically western diet, WD). A one-way Anova for repeated measurements was used.

**Results:**

No significant differences were detected between VLCKD and WD in all strength tests. Significant differences were found in body weight and body composition: after VLCKD there was a decrease in body weight (from 69.6 ± 7.3 Kg to 68.0 ± 7.5 Kg) and fat mass (from 5.3 ± 1.3 Kg to 3.4 ± 0.8 Kg p < 0.001) with a non-significant increase in muscle mass.

**Conclusions:**

Despite concerns of coaches and doctors about the possible detrimental effects of low carbohydrate diets on athletic performance and the well known importance of carbohydrates there are no data about VLCKD and strength performance. The undeniable and sudden effect of VLCKD on fat loss may be useful for those athletes who compete in sports based on weight class. We have demonstrated that using VLCKD for a relatively short time period (i.e. 30 days) can decrease body weight and body fat without negative effects on strength performance in high level athletes.

## Background

Many procedures used for body weight reduction by athletes in sports that include weight categories lead to a series of negative side effects which directly influence physiological efficiency during sports performance. The practice of rapidly losing a significant amount of weight, through low calorie diets, deliberate dehydration, saunas etc., just before competition, is widespread [1-3]. These traditional methods are often unsafe and typically impair health, physiological function, water balance, electrolytes, glycogen and lean body mass [1,4-6] and are sometimes illegal as with the use of diuretics [3].

However for athletes competing in sports divided into weight categories a safe method of weight loss that does not impair performance can be a legitimate and important tool. For example, bodybuilders regularly need to reduce fat and/or weight before competition preferably without affecting muscle strength or muscle size [7] and a VLCKD (very low carbohydrate ketogenic diet) is commonly used to achieve this. VLCKD is a diet in which the daily carbohydrate intake is below 30 g and this restriction limits glucose availability to tissues, stimulating ketogenesis in the liver. The physiological function of ketosis is to supply the heart and central nervous system (CNS) with a high energy metabolic substrate during reduced glucose availability – by this mechanism ketones allowed our ancestors to survive and remain efficient even when deprived of food [8,9]. On this basis the ketosis induced by a VLCKD may be defined as “physiological ketosis” to distinguish it from the severe pathological ketosis (or ketoacidosis) commonly seen in uncontrolled diabetes [10-12]. The use of low carbohydrate ketogenic diets for weight loss, despite their efficacy, has been an area of controversy. In the last few years though an increasing amount of evidence has accumulated concerning the positive effects on short term weight loss, metabolic profile with regards to insulin sensitivity, glycemic control and serum lipid values [12-16]. These effects appear potentially very attractive for athletes needing to lose fat mass quickly but, curiously, despite the huge amount of scientific literature about ketogenic diets, their influence on sport performance remains poorly investigated. Recently Kreider and colleagues studied the effect of a specific exercise program in overweight woman with a VLCKD or normal carbohydrate content diet [17], but only few papers that focus specifically on the influence of VLCKD on sports performance have been published, and with conflicting results: showing benefits [18,19], no effect [20,21] or impairment [22,23].

The present study set out to investigate if a VLCKD could be useful for athletes, especially for those engaged in sports involving weight categories where weight loss without negative changes in the body composition (i.e. loss of muscle mass) and performance is often needed. To the best of our knowledge no previous study has investigated the influence of a VLCKD on strength performance and on explosive strength performance in competitive athletes.

## Methods

### Subjects

Nine high-level male athletes (age 21 ± 5.5), elite artistic gymnasts, were recruited for this study. Subjects competed in the Italian premier league for the CorpoLibero Gymnastics Team ASD, Padova, Italy and include two athletes belonging to the Italian national team. The mean volume of weekly training was about 30 hours. During the VLCKD period (30 days) the athletes were asked to keep to their normal training schedule. During a preliminary meeting it was explained that during the first three weeks it was necessary to almost totally exclude carbohydrates and a detailed menu containing permitted and non-permitted foods was provided to each participant, along with the components of the ketogenic diet with phytoextracts diet described below. All gymnasts read and signed an informed consent with the testing procedures approved by the council of the Human Anatomy and Physiology Department, University of Padova.

### Experimental design

**Figure 1:**
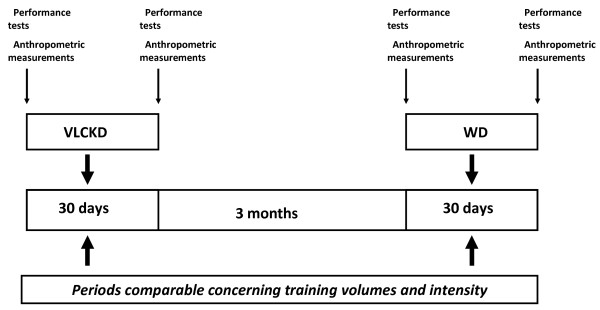
Scheme of the experimental protocol.

Subject measurements were taken, according to the methodology described below, before starting the VLCKD and repeated after thirty days of VLCKD. Since we chose a within subject design to strengthen the study (Subjects served as their own control), the athletes were re-tested during a second training period comparable in terms of intensity and volume of training to the first one.. The work load between athletes was similar because the team training regimes are strictly controlled, and recorded, due to the elite nature of their competition. The protocol took place three months later to ensure a comparable training load and achieve this goal the intensity and volume of training during the two periods (hours of training, kind of exercises, etc.) was carefully measured. During the second experimental session the subjects followed their normal diet (WD) instead of the VLCKD. The test procedure before and after WD was the same as the first testing session (Figure 1). As in the experimental period also during the control period the athletes were invited to continue their normal training. One subject withdrew from the study due to injury.

### Diet

Before the start of the study, each athlete was given a detailed list containing the foods permitted and prohibited in a ketogenic diet. The diet consumed was primarily made of beef and veal, poultry, fish, raw and cooked green vegetables without restriction, cold cuts (dried beef, carpaccio and cured ham), eggs and seasoned cheese (e.g. parmesan). The drinks allowed were infusion tea, *moka* coffee and the herbal extracts. The foods and drinks that athletes avoided included alcohol, bread, pasta, rice, milk, yogurt, soluble tea and barley coffee. In addition to facilitate the adhesion to the nutritional regime, each athlete was given a variety of speciality meals constituted principally of protein and fiber These meals (TISANOREICA® by Gianluca Mech SpA, Asigliano Veneto, Vicenza, Italy) which are composed of high quality protein (equivalent to 18 grams/portion) and virtually zero carbohydrate (but that mimic their taste) were included in the standard ration [16,24].

Both the foods mentioned in the list and the standard ration could be consumed during the same meal and VLCKD was taken by athletes *ad libitum.* During the VLCK diet, the athletes also consumed some specific herbal extracts: 20 ml of extract A, 20 ml of extract B and 50 ml of extract C as described in Tables 1 and 2. Moreover, during ketogenic diet periods, athletes assumed 1 caplet in of a multivitamin-mineral supplement each morning ([19,25,26]. The composition of the caplets was: Magnesium19 mg, Calcium 16 mg, Phosphorus 8 mg, Zinc 4.5 mg, Iron 4.62 mg, Manganese 1 mg, Potassium 0.5 mg, Copper 0.4 mg, Chromium 28.55 μg, Selenium 4 μg, Niacin 10 mg, Beta carotene 1.8 mg, Folic Acid 66 μg, Biotin 30 μg, Vitamin C 19.8 mg, Vitamin E 3.3 mg, Pantothenic Acid 1.98 mg, Vitamin B6 0.66 mg, Vitamin B2 0.53 mg, Vitamin B1 0.426 mg, Vitamin D3 1.65 μg, Vitamin B12 0.33 μg (Multivitaminico Balestra e Mech, Gianluca Mech SpA, Asigliano Veneto VI).

**Table 1:** Plant extracts used during the VLCKD

**Plant extracts**		**Composition**
Extracts A, ml/day	20	Durvillea antarctica, black radish, mint, liquorice, artichoke, horsetail, burdock, dandelion, rhubarb, gentian, lemon balm, chinaroot, juniper, spear grass, elder, fucus, anise, parsley, bearberry, horehound
Extracts B, ml/day	20	Serenoa, Red clover, Chervil, Bean, Elder, Dandelion, Uncaria, Equisetum, Horehound, Rosemary
Extracts C, ml/day	50	Horsetail, asparagus, birch, cypress, couch grass, corn, dandelion, grape, fennel, elder, rosehip, anise
Extracts D, ml/day (only weeks 1 and 2)	40	Eleuthero, eurycoma longifolia, ginseng, corn, miura puama, grape, guaranà, arabic coffee, ginger

**Table 2:** Main actives ingredients of phytoextracts and their reported beneficial effects

**Extract**	**Main Active ingredients**	**Reported beneficial effects**	**Refs**
A	Mint	- indigestion	[25]
	black radish	- antioxidant	[26]
	burdock	- choleretic, increases bile secretion helping digestion	
B	Serenoa Repens (saw palmetto)	hormonal regulating effects	[27]
	White bean	alpha-amylase inhibitory properties and has been reported to aid weight loss and glycemic control	[28]
			[29]
C	Equisetum	Antioxidant	[30]
	Dandelion (Taraxacum officinale	diuretic	[31]
		glycemic control	[32]
D	Ginseng	Ameliorate the commonly reported symptoms of weakness and tiredness during the 1^st^ phase of ketosis (1/2 weeks)	[33]
	Miura Puama		[34]
	Guaranà		[35]

During the second period of the study, the athletes themselves assumed the function of a control group and consumed their usual diet that is very close to the present US diet western diet (WD) [38] with the exception of fat origin (monounsaturated fats, i.e. olive oil in our subjects vs. saturated fats in the typical US diet). The diet consumed was composed mainly of potatoes, whole grains (bread, pasta, whole wheat, rice, and potatoes), meat, fish, eggs, poultry, vegetables, legumes, fruits, condiments (mainly olive oil), whole milk and wine. As with the ketogenic diet, the WD was taken *ad libitum* according to the nutritional habits of the athletes.

The diets were explained to all subjects by a qualified dietician during an individual visit. Dietary intake was measured by validated 3- day food diary [39] that has been used in the past in studies with athletes [40] and analysed by Dietnext® (Caldogno, Vicenza, Italy) software. During the ketogenic period the prescribed daily intake of carbohydrate was 22 g. The percentage distribution of total daily energy macronutrients was 54.8% fat, 40.7% protein and 4.5% carbohydrates. The total amount of daily kilojoules was 8254.5 ± 1136. During the WD period the macronutrients were distributed in the following order: 46.8% carbohydrate, 38.5% lipids, 14.7% protein. The Western diet provided a total daily kJ 9520.7 ± 1080.71 (Table 3).

**Table 3:** Average macronutrient and total energy intake during the ketogenic and the free diet period

	**VLCKD**	**WD**
**CHO g**	22 ± 2.3	266.1 ± 30.8
**PRO g**	200.8 ± 18.3	83.5 ± 9
**FAT g**	120.2 ± 29.5	97.3 ± 14.4
**CHO KJ**	368.5 ± 38.4	4455.6 ± 515
**PRO KJ**	3363 ± 306.5	1398.5 ± 150.1
**FAT KJ**	4523 ± 1112.8	3666.6 ± 543.8
**CHO % KJ tot**	4.5 ± 0.5	46.8 ± 2.1
**PRO % KJ tot**	40.7 ± 5.7	14.7 ± 1.1
**FAT % KJ tot**	54.8 ± 6.0	38.5 ± 2.6
**KJ Tot**	8254.5 ± 1136.0	9520.7 ± 1080.7

Data are expressed as mean and SD.

### Measurements

Before and after the diet period, muscle and fat amounts and percentages, were assessed by skinfold measurement which are highly related to percent body fat in fit and healthy young men [41-43]. We used software (Fitnext®, Caldogno, Vicenza, Italy) that includes 9 skinfolds (triceps, biceps, pectoral, subarmpit, subscapular, iliac crest, mid-abdominal, anterior thigh, medial calf), 6 bone circumferences (arm, forearm, waist, hip, thigh, calf), 4 bone diameters (elbow, wrist, knee, ankle), waistline and hip circumference measurement [24,44]). Anthropometric measurements were performed according to the Anthropometric Standardization Reference Manual [45]. Weight was measured to the nearest 0.1 kg using an electronic scale (Tanita BWB-800 Medical Scales, USA), and height to the nearest 1 cm using a Harpenden portable stadiometer (Holtain Ltd, UK). Skinfolds were measured to the nearest 1 mm using a Holtain caliper (Holtain Ltd, UK), and circumferences to the nearest 0.001 m using an anthropometric tape. All measurements were taken by the same operator (LC) before and during the study according to standard procedures [45,46].

Following the anthropometric assessment a standardized warm-up lasting 15 minutes consisting of callisthenic exercise was carried out. After 5–8 minutes all the athletes underwent the following strength tests: squat jump (SJ), counter movement jump (CMJ), 15 seconds of consecutive CMJs, push-ups test, reverse grip chins test, legs closed barrier maximum test, parallel bar dips test. Jump tests were performed on a contact mat (Ergojump—Bosco system, srl, S. Rufina di Cittaducale, Rieti, Italia), that allowed the measurement of height of jump, time of flight and time of contact. The height of jumps was calculated according to the Asmussen and Bonde-Petersen formula [47]. All jump test techniques assume that the athlete’s position on the mat is the same both at take-off and landing. During jumps athlete’s hands were kept on hips to minimize upper limbs contribution and trunk was maintained erect. The SJ test was performed from the seated position maintained at least for 1 second (knee secured at 90° of knee flexion) then athletes were asked to jump. The CMJ starting from a standing position, then subjects were instructed to perform a rapid downward movement to about 90° of knee flexion immediately followed by an upward movement. The CMJs were consecutively repeated during 15 seconds without recovery between jumps. For CMJs mean jump height and mechanical power per kilogram of body weight were computed [48]. For all three test types the subjects were requested to jump as high as possible. SJ and CMJ were performed three times with two minutes rest between each trial. The best performance was retained and included in the test [49]. The exercises for the upper part of the body were carried out by each athlete until exhaustion. In the push-up test the subjects were positioned with the palms of the hands in support on the floor at shoulder width; at the start of the exercise, the subjects folded their arms while contemporaneously lowering the trunk to the floor.

In the reverse grip chins test the athletes grabbed the bar (as used in artistic gymnastics) at shoulder width; the subjects first brought the chest to the bar height. In the legs closed barrier maximum test, the subjects grab the bar and without oscillating the pelvis elevated the lower limbs to bring the back of both feet in contact with the bar. During parallel bar dips test the subjects lowered themselves to the limit allowed by the shoulder joint.

Test-retest reliability for all exercises obtained in our setting was consistent with previous findings: ICCr: SJ O.97, CMJ 0.99, push-up 0.98, reverse grip chins 0.96, leg closed barrier 0.90, parallel dips 0.95 [50-55].

### Statical analysis

A one-way Anova for repeated measurements was used with significance placed at p < 0.05. When appropriate a Bonferroni post hoc test was used to compare selected data.

## Results

**Figure 2:**
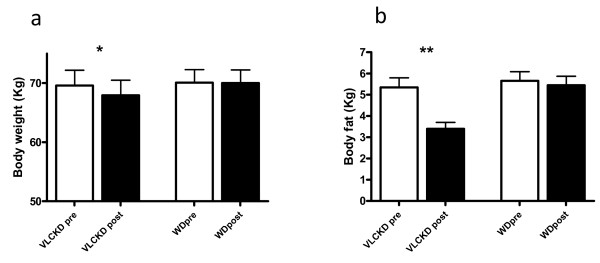
**Changes in body weight (a) and kilograms of fat (b) before and after very low carbohydrate diet and western diet.** SD are showed with bars.

No significant differences in anthropometric variables or in athletic performance were detected at basal conditions before either experimental trial. There was a significant difference pre and post VLCKD in body weight (from 69.6 ± 7.3 Kg to 68.0 ± 7.5 Kg p < 0.05) (Figure 2a), fat mass (from 5.3 ± 1.3 Kg to 3.4 ± 0.8 Kg p < 0.001) (Figure 2b), fat percentage (pre 7.6 ± 1.4; post 5.0 ± 0.9; P < 0.001) and lean body mass percentage (from 92.4 ± 1.44 to 95.0 ± 1.0; P < 0.001) whilst there was no significant difference comparing pre and post WD. Moreover after VLCKD muscle mass (pre 37.6 Kg ± 3.9; post 37.9 Kg ± 4.5) and lean body mass (pre 64.2 ± 6.5; post 64.6 ± 7.1) remained substantially constant (Table 4).

**Table 4:** Performance, anthropometric and body composition results befor and after diet intervention

	**VLCKD start**	**VLCKD end**	**WD start**	**WD end**
**performance results**				
**SJ**	0.42 ± 0.04	0.42 ± 0.05	0.41 ± 0.04	0.40 ± 0.04
**CMJ**	0.45 ± 0.04	0.43 ± 0.05	0.43 ± 0.06	0.43 ± 0.05
**reverse grip chins**	17 ± 4.2	16.6 ± 4.6	15.2 ± 3.4	15.2 ± 5.8
**push-ups**	36 ± 6.3	38.8 ± 4.7	37 ± 11.8	43.5 ± 18.1
**legs closed barrier**	19.2 ± 4.96	21.7 ± 6.35	17.2 ± 5.0	16 ± 4.77
**parallel bar dips**	25.8 ± 8.35	28.2 ± 9.31	23 ± 12.19	27 ± 10.61
**Anthropometric and body composition results**				
**muscle Kg**	37.6 ± 3.9	37.9 ± 4.5	38.4 ± 4.1	38.6 ± 4.5
**Fat Kg**	5.3 ± 1.3	3.4 ± 0.8 **	5.1 ± 1.3	4.9 ± 1.1
**fat %**	7.6 ± 1.4	5.0 ± 0.9 **	8.0 ± 1.3	7.7 ± 1.2
**Lean body mass Kg**	64.2 ± 6.5	63.1 ± 7.1	61.5 ± 4.3	61.8 ± 4.6
**lean body mass %**	92.4 ± 1.4	95.0 ± 1.0 **	92.0 ± 1.3	92.3 ± 1.2
**Weight**	69.6 ± 7.3	68.0 ± 7.5 **	70.1 ± 6.2	70.0 ± 6.3

Data are espresse as mean and SD. Symbols: ** = p < 0.001 significant difference from baseline; * = p < 0.05 significant difference from basline.

As can be seen in Table 4 there were no significant differences in any performance tests before and after VLCKD nor before and after WD.

## Discussion

The aim of our research was to verify the effects of a VLCKD on power strength performance in elite athletes. It is well known that VLCKD’s promote weight loss very rapidly [56]. Surprisingly, despite the various unhealthy procedures for body weight reduction (low calorie diets, deliberate dehydration, saunas, pharmacological methods) that are in very widespread use by athletes, especially those competing in sports with weight categories, only few studies examining VLCKD and exercise have been published. Phinney [19] found that in moderately obese, untrained subjects a prolonged exercise at 60% of VO_2_max can be sustained in the virtual absence of dietary carbohydrate (<10 g/d) for 6 wk with a surprising increase in treadmill time duration of 155% respect to baseline (from 168 to 259 minutes). In a second study [57], Phinney studied the effect of chronic ketosis on exercise performance in endurance-trained athletes finding that aerobic endurance exercise by well-trained cyclists was not compromised by four weeks of ketosis. In contrast White suggested that VLCKD enhanced perception of fatigue during a 90 min walk, but in this study only RPE (Rate of Perceived Exertion) was significant whilst average heart rate and exercise intensity expressed at %HR max did not change. Unfortunately other performance indexes such as VO_2_max and blood lactate were not investigated [22]. More recently a broader study [18] reported that a ketogenic diet enhanced fat oxidation without detrimental effects on maximal or submaximal markers of aerobic exercise performance in obese subjects. Interestingly, to our knowledge, this is the first study published that measured the effects of VLCKD on strength performance and the authors reported no difference in strength isometric performance between VLCKD group and high carbohydrate group.

Three factors should be taken into account to explain these conflicting results: 1) the time needed for keto-adapatation (approximately 7 days), 2) usage or not of electrolyte supplementation 3) the protein intake. According to the first factor, most studies have maintained the VLCKD for less than two weeks, which not sufficient to accomplish the full ketogenic metabolic adjustment (since 7 days are required for keto-adaptation leaving just a few days to see the effects of ketosis during these short dietary protocols). In our experimental design the ketogenic period was maintained for 30 days. Regarding adequate electrolyte supplementation it is noteworthy that a supplement containing sodium and potassium is needed to maintain an effective nitrogen balance with functional tissue preservation [58] and the Tisanoreica® protocol reported here included an electrolyte supplementation [16]. Finally to maintain lean body mass a protein intake of 1.2–1.7 g/kg/bw with reference to body weight is required [58]. Most techniques used for weight loss in sports lead to a reduction of lean body mass with consequent negative effects on performance. The effects of the reduction in daily protein intake below 1.2 g/kg/bw during a VLCKD, includes the gradual loss of lean tissue and therefore the loss of physical performance as demonstrated by Davis [59]. The daily intake of protein during the ketogenic phase in our study was approximately 2.8 g/kg (assuming an increased protein requirement due to the very intense physical activity) [60,61]. White et. al., provided 30% of caloric intake from protein [22] and basing calculations on a previous report by the same group [62], provided a daily intake of 125 g, probably not enough to provide the necessary amount of amino acids for gluconeogenesis. It is known that for the preservation of muscle and an adequate level of physical performance during a restricted diet a minimum of 135 g of protein per day is necessary for a subject of 80 kg. Eaton suggests that in ancestral humans, protein provided about 30% of daily energy intake (which corresponds to an intake of approximately 3 g/kg per day for a 70 kg individual consuming 12 500 kJ (3000 kcal)/d [63]. In our study, it can be observed that despite a significant decrease of fat percentage and fat absolute amount, the strength performances remained stable after 30 days of VLCKD. Recently we have summarized the factors involved in the fat loss effect of VLCKD diets [12]:

1. Satiety effect of proteins leading to appetite reduction in which also ketone bodies may have a role, although the mechanism is not clear;

2. >Reduction in lipid synthesis and increased lipolysis mechanisms;

3. Reduction in at rest respiratory quotient and therefore an increase in fat metabolism for energy use;

4. Increased metabolic expenditure caused by gluconeogenesis and the thermic effect of proteins.

The maintenance (or strictly speaking the visible increase, albeit not significant) of the amount of lean body mass, muscle and percentage of muscle during the period of VLCKD needs to be underlined and this muscle sparing effect can be explained through the mechanism of ketosis. As stated before, fatty acids which are normally used as a major fuel for some tissues such as muscle, cannot be used by the CNS because they cannot cross the blood–brain barrier. During starvation (fasting) this becomes a problem, particularly for organisms such as humans in which CNS metabolism constitutes a major portion of the resting basal metabolic rate (~20%). During the initial fasting period our body provides glucose for the metabolic needs of the CNS via break down of muscle tissue to provide the amino acid precursors for gluconeogenesis. Obviously the organism could not survive long under such wasting conditions and ketone bodies (KB) therefore represent an alternate fat-based fuel source that spares muscle protein [12]. It is noteworthy that the mechanism underlying the increase of body fat utilization has some pathways in common with mechanisms contributing to the lack of muscle mass increase. The use of FFA and ketones for muscle fuel spares muscle protein and is thus anti-catabolic. During the ketogenic period, whilst blood glucose decreases by a small amount, remaining at around 80–90 mg/dl, insulin remains at very low levels (7 mU/L) [58,64,65]. Insulin is involved in increased liposynthesis and decreased lipolysis so a reduction in insulin levels facilitates mobilization from fat stores; on the other hand insulin is fundamental for the muscle growth pathway (via IGF-1, mTOR, AKT etc.). Our data confirm that during the ketogenic diet it is actually very difficult to increase muscle mass and therefore the maintenance of muscle mass, the lack of lean mass loss, may be considered a successful objective for our athletes. We noted that some athletes complained that they were not able to finish the exercises proposed during the training but these were temporary effects present only during the first week after which they disappeared completely. One of the limits of our research is the low sample number due to the common problem of recruiting high level athletes for experimental protocol during the competitive season. It is possible to conclude though that physical performance was not altered in these well-trained individuals using an iso-caloric low-CHO diet (<20 g·d^−1^ CHO) with an adequate vitamin, minerals and protein (2.8 g · kg^−1^ · d^−1^) supply, compared to a normal diet.

## Conclusions

Many coaches do not favorably accept the use of a ketogenic diet by their athletes, both due to the absence of knowledge of the effects of the LCKD and due to fear that the diet can rebound on the physical performance of the athlete. Unfortunately there are very few studies on the topic “ketogenic diet and exercise”, showing consistent methods and results. Those that reported negative effects of VLCKD on performance were only carried out for a time of up to 15 days [22]; but a longer period of time is necessary in order to induce the keto-adaptation [66]. This process of keto-adaptation seems to require a significant adherence to the dietary restriction of carbohydrate that needs to last at least 10/14 days to produce the positive reported effects. Individuals who intermittently consume carbohydrates during a ketogenic diet reduce their tolerance to exercise [18,19,22,58]. Our data suggest that athletes who underwent a VLCKD with adequate protein intake lost weight and improved body composition without any negative changes in strength and power performance. Taken together these results suggest that a properly monitored and programmed ketogenic diet could be a useful, and safe, method to allow the athletes to reach their desired weight categories without the unnecessary and harmful procedures currently in use. In conclusion, this dietetic approach in the short term could be helpful in sports that involve weight categories.

## Competing interests

This work was partially funded by GianlucaMechSpA, Orgiano (VI), Italy. GianlucaMechSpA AP and LC research activity is funded by dept. of Human Anatomy and Physiology, University of Padova; KG research activity is funded by the Biomedical Engineering Laboratory, Institute of Communication and Computer Systems, National Technical University of Athens, Athens, Greece. AP has been a consultant for and has received grant/research support from Gianluca Mech Spa. LC is scientific consultant for Gianluca Mech SpA, Asigliano Veneto (VI), Italy. The other authors declare no competing interests. Investigators conducted the study in its entirety and maintained exclusive control of all data and analyses. The funding source had no involvement in any part of the recruitment of participants, study intervention, data collection, data analyses, interpretation of the data, or preparation or review of this manuscript.

## Authors’ contributions

A Paoli was the main researcher and was responsible for study design, statistical analysis and interpretation of data and draft of manuscript, conceived the study, participated in its design, drafted the manuscript and performed the statistical analysis. KG was responsible for analysis and interpretation of data and helped to draft the manuscript. D D’A participated in the study design and coordination and helped to draft the manuscript. CB was responsible for study design and acquisition of data. LC was responsible for diet prescription and analysis. TM helped to draft the manuscript. AB participated in the design of the study and in the statistical analysis. A Palma participated in the design of the study and helped to draft the manuscript. All authors read and approved the final manuscript.
